# Supplementary material for: The interactome of intact mitochondria by cross-linking mass spectrometry provides evidence for coexisting respiratory supercomplexes
Source: Mol Cell Proteomics. 2017 Dec 8;17(2):216–32. doi: 10.1074/mcp.RA117.000470 (PMC5795388; doi:10.1074/mcp.RA117.000470)
Supplement: Supplemental Data [file supp_RA117.000470_133922_0_supp_23978_fzffwf.zip › spectra_annotation/mito_spectra_annotation/381-1-10-1-29-1.pdf]

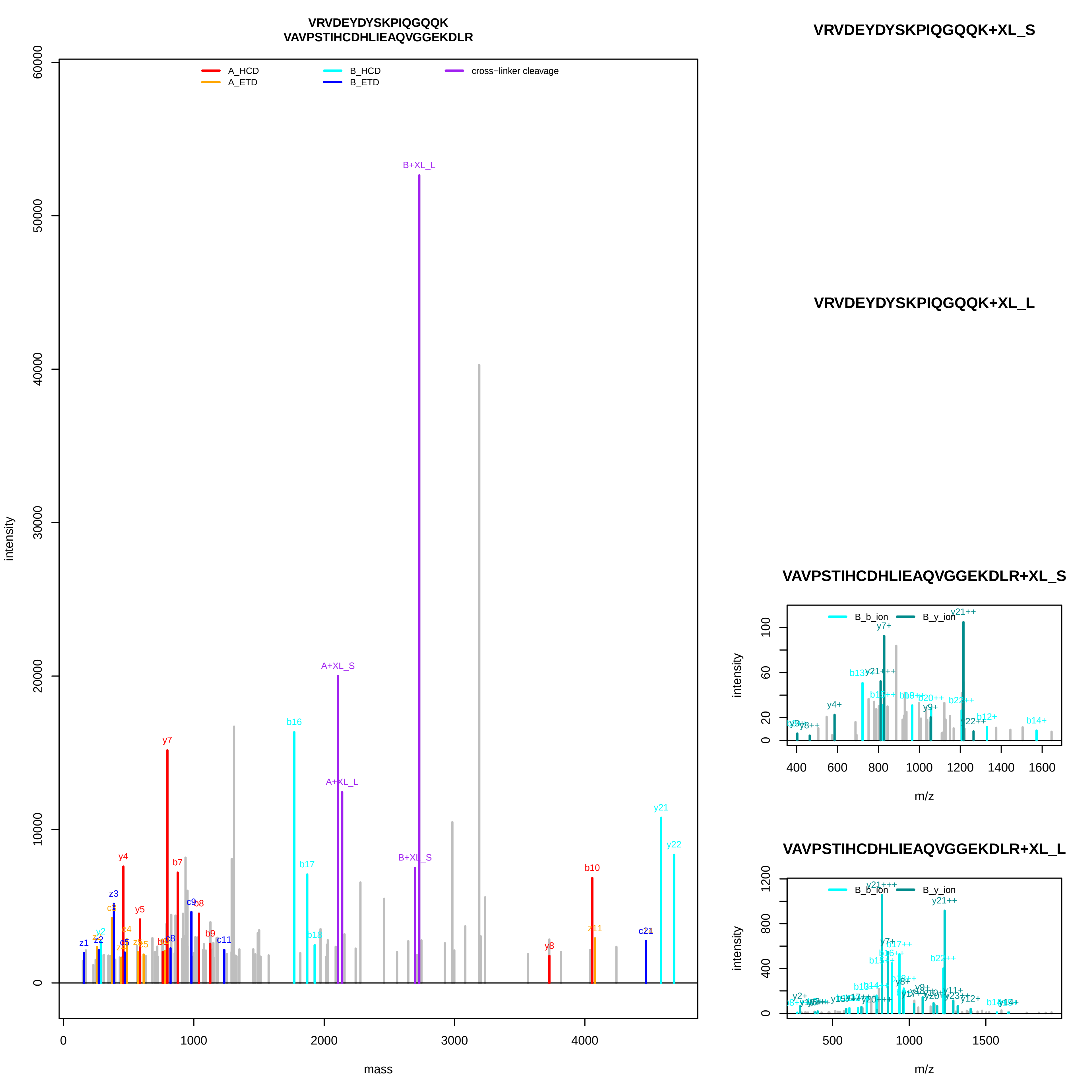
VRVDEYDYSKPIQGQQK
VAVPSTIHCDHLIEAQVGGEKDLR
A_HCD
A_ETD
B_HCD
B_ETD
cross−linker cleavage
intensity
mass
0
10000
20000
30000
40000
50000
60000
1000
2000
3000
4000
B+XL_L
A+XL_S
A+XL_L
B+XL_S
y7
y4
b7
y5
b8
b9
z3
c9
c11
b16
b17
b18
y8
b10
z11
c21
y21
y22
VRVDEYDYSKPIQGQQK+XL_S
VRVDEYDYSKPIQGQQK+XL_L
VAVPSTIHCDHLIEAQVGGEKDLR+XL_S
B_b_ion
B_y_ion
y21++
y7+
y4+
y9+
b12+
b14+
m/z
400
600
800
1000
1200
1400
1600
VAVPSTIHCDHLIEAQVGGEKDLR+XL_L
y21+++
y21++
b22++
500
1500
